# Effect of Biologic Therapies in Treating Moderate-to-Severe Ulcerative Colitis: A Systematic Review and Meta-Analysis

**DOI:** 10.7759/cureus.85923

**Published:** 2025-06-13

**Authors:** Ooha Thadiboina, Syed Saim Ali Shah, Rubela Ray, Sarah A Hack, Mahpara Munir, Qalandar Shah, Amritveer Bhullar, Syed Zargham Hussain Shah, Mohammed Abdul Muhaimin Ali, Uzma Nureen, Sana Afzal, Izzat Izzat

**Affiliations:** 1 Internal Medicine, Osmania Medical College, Hyderabad, IND; 2 Anesthesia, Chaudhry Pervaiz Elahi (CPE) Institute of Cardiology, Multan, PAK; 3 Internal Medicine, Bankura Sammilani Medical College and Hospital, Bankura, IND; 4 Medicine, St. George’s University, St. George’s, GRD; 5 Internal Medicine, Hurley Medical Center/Michigan State University (MSU), Flint, USA; 6 Surgery, Bahria University of Health Sciences, Karachi, PAK; 7 Internal Medicine, D. Y. Patil Medical College, Kolhapur, IND; 8 Medicine, Frontier Medical and Dental College, Abbottabad, PAK; 9 Medicine, Osmania Medical College, Hyderabad, IND; 10 Internal Medicine, Akhtar Saeed Medical and Dental College, Lahore, PAK; 11 Internal Medicine, Lahore Medical and Dental College, Lahore, PAK; 12 Modern Medical Education, Caribbean Medical University (CMU), Yonkers, USA

**Keywords:** a systematic review, biologic therapies, clinical remission, meta-analysis, ulcerative colitis (uc)

## Abstract

Biological therapies have emerged as effective treatments for moderate-to-severe ulcerative colitis (UC). This systematic review and meta-analysis aimed to assess the efficacy of different biologic agents for inducing clinical response, remission, and mucosal healing in patients with moderate-to-severe UC. A systematic literature search was conducted in PubMed, Excerpta Medica database (EMBASE), and Cochrane Library from inception to February 2025. Randomized controlled trials (RCTs) and prospective cohort studies evaluating biologics in adults with moderate-to-severe UC were included. The primary outcomes were clinical response, clinical remission, and mucosal healing. Random-effects meta-analyses were performed to calculate pooled effect estimates. Forty-three studies were included. Biologics were significantly more effective than placebo for inducing clinical response rates (odds ratio (OR): 2.19 (CI 95%: 2.66-3.19) p<0.00001, I^2^= 83%), remission rates (OR: 3.10 (CI 95%: 2.82-3.42) p<0.00001, I^2^= 92%), and mucosal healing (OR: 1.66 (CI 95%: 1.47-1.88) p<0.00001, I^2^= 85%) among UC patients. Heterogeneity was significant for most outcomes (I² > 50%). The quality of evidence ranged from low to moderate. Biologic therapies are effective for inducing response, remission, and mucosal healing in moderate-to-severe UC. Further high-quality studies are needed to directly compare different biologics and evaluate long-term outcomes.

## Introduction and background

Ulcerative colitis (UC) is a chronic inflammatory bowel disease (IBD), characterized by persistent colonic mucosal inflammation, which often starts in the rectum and spreads to all parts of the colon [1]. With incidence rates ranging from nine to 20 cases per 100,000 person-years, UC has historically been more common in Western countries, e.g., North America and Northern and Western Europe [2]. However, newly industrialized regions such as Asia, the Middle East [3], and South America [4] have seen a significant rise in newly diagnosed cases in recent decades. Urbanization, nutritional changes, environmental changes, and better disease detection are all major causes for this rapid increase in the incidence of UC [5].

Its origin is complex and includes changes in gut microbiota, immunological dysregulation, genetic predisposition, and environmental factors [6, 7]. Rectal bleeding, diarrhea, abdominal pain, and urgency are prominent symptoms of UC that seriously lower quality of life. Moderate-to-severe instances are characterized by persistent disease activity that is often resistant to standard treatments such as immunomodulators, corticosteroids, and aminosalicylates [8].

Over the past 20 years, the treatment of moderate-to-severe UC has changed due to the introduction of biological treatments [9]. Complex, protein-based medications known as biologics are made from living cells and are intended to target particular immune system elements that contribute to inflammation [9, 10]. Based on encouraging outcomes from clinical studies, they include integrin receptor antagonists (vedolizumab) [11], interleukin inhibitors (ustekinumab) [12], and tumor necrosis factor-alpha (TNF-α) inhibitors (infliximab, adalimumab, and golimumab) [13] that have been approved for the treatment of UC. These medications seek to lessen corticosteroid reliance, encourage mucosal repair, and result in sustained clinical remission [14, 15].

Outcomes of biological treatments can differ depending on patient heterogeneity, illness severity, immunogenicity, and loss of response over time, even with the growing availability of biologics [10, 16]. There is still ongoing research into the relative safety and efficacy of various drugs, particularly in light of new randomized clinical trials (RCTs) and empirical data. Furthermore, real-world research is crucial for comprehending how these therapies function in varied populations with comorbidities, concurrent medications, and varying adherence levels, even while clinical trials offer efficacy data under ideal circumstances.

Various publications have reported the effectiveness of vedolizumab, tofacitinib, infliximab, and golimumab individually [17-20]. No study has reported the outcomes of all biological drugs comprehensively to suggest a drug with more effective outcomes. Furthermore, clinical professionals find it difficult to choose the best biologic for each patient due to the therapeutic arsenal’s quick increase. Clinical guidelines and evidence-based decision-making require a thorough synthesis of the available data. An updated meta-analysis that systematically analyzes the efficacy of numerous biologics in moderate-to-severe UC is necessary, especially in light of recently published studies and long-term outcome data, even though prior evaluations have focused on individual biologic agents or certain outcomes.

The purpose of this systematic review and meta-analysis is to assess how well biologic treatments work for treating moderate-to-severe UC. This review aims to provide a consolidated evidence base to guide therapeutic strategies for patients who are not responding to conventional therapy or who need maintenance of long-term disease control by combining data from high-quality observational studies and RCTs.

## Review

Methods

Search Design

This systematic review and meta-analysis were performed by following the Preferred Reporting Items for Systematic Reviews and Meta-Analyses (PRISMA) guidelines [21] to fulfill research aims. There was no need for an additional ethical review due to the involvement of previously published retrospective and prospective cohort studies.

Population Intervention Control Outcome (PICO) Framework

This study used the PICO framework to guide the search: P: Patients with moderate-to-severe UC; I: Biologic therapies (e.g., anti-TNF agents, anti-integrins, anti-IL agents, JAK inhibitors); C: placebo or conventional therapies (e.g., corticosteroids, immunomodulators) or head-to-head comparisons between different biologics; O: clinical remission, clinical response, mucosal healing, and adverse events.

Search Strategy

The PRISMA guidelines assisted in the selection of research articles related to the study aims. Three electronic databases, PubMed, Excerpta Medica database (EMBASE), and the Cochrane Library, were searched from inception to February 2025. The Medical Subject Headings (MeSH) keywords used for the search of research articles from PubMed were ("Ulcerative Colitis"[MeSH] OR "Inflammatory Bowel Diseases"[MeSH]) AND ("Biological Products"[MeSH] OR "biologic therapy" OR "Anti-TNF agents" OR "JAK inhibitors") AND ("Treatment Outcome"[MeSH] OR "Effectiveness" OR "Clinical Remission") AND ("Moderate-to-Severe" OR "moderate" OR "severe"). A similar search strategy was used for other databases. The databases were searched from January 2011 to April 2025. The search was restricted to the English language. We carefully examined the reference lists of all previous systematic reviews and meta-analysis-based articles to search for further research articles.

Eligibility Criteria 

The eligibility criteria were used to select and screen research articles after searching for research articles from electronic databases.

Inclusion Criteria

Studies were included if they analyzed adult patients over 18 years of age diagnosed with moderate to severe UC and evaluated the effects of biological therapies compared to placebo. Eligible studies reported outcomes such as response rates, remission rates, and mucosal healing. Only primary research studies, including RCTs and prospective cohort studies, were considered. Additionally, studies were required to have full-text availability and be published in the English language.

Exclusion Criteria

Studies were excluded if they involved patient populations with other types of cancer or focused on individuals receiving therapies other than biological treatments. Research investigating the long-term outcomes or maintenance effects of biological drugs for UC was also excluded. Non-primary studies, such as systematic reviews, meta-analyses, comprehensive reviews, narrative reviews, case-control studies, and editorials, were not included. Finally, studies published in languages other than English or lacking full-text access were excluded.

Data Extraction

Two independent reviewers extracted the data to be placed in a pre-specified table. The studies obtained by the database search were entered into the EndNote library (Clarivate, London, UK). Duplicates were excluded in the next step. The eligibility criteria were applied by reviewers in a blinded manner to all individual studies. Discrepancies were sorted by mutual agreement. Data related to demographic information, such as authors, year, country, study population, study design, study follow-up, and primary outcomes, were extracted. Discrepancies were resolved by consulting with a third reviewer.

Risk of Bias Assessment

The Cochrane Risk of Bias tool was applied to assess the risk of bias of included RCTs. The risk bias of included studies was evaluated on the basis of six domains: allocation concealment, blinding of participants, selection bias, blinding of outcome assessment, selective reporting, and other bias. The score or level of each included study was categorized into low risk, unclear, and high risk [22].

Quality Assessment of Cohort Studies

The quality of the included studies was assessed by using proper tools on the basis of study design. Due to the inclusion of observational studies, the Newcastle-Ottawa Scale (NOS) was used for quality assessment [23]. The score of >7 for included studies was considered low risk, scores of five to seven for included studies indicated moderate risk, and <5 for included studies showed high risk. Any disagreement in risk bias assessment was resolved through consensus.

Statistical Analysis

Review Manager Software (The Cochrane Collaboration, 2020, Review Manager (RevMan) (computer software) (version 5.4) was used to conduct statistical analyses. The studies in the analysis are assumed to be a random sample from a universe of potential studies, and this analysis was used to make an inference about that universe. Pooled analysis of data was performed for studies with potential heterogeneity using random effects models. Statistical significance was set at P < 0.05 and was considered statistically significant [24]. Heterogeneity was evaluated using the I² statistic, with I² values > 50% indicating significant heterogeneity.

Results

Search Results

**Figure 1:**
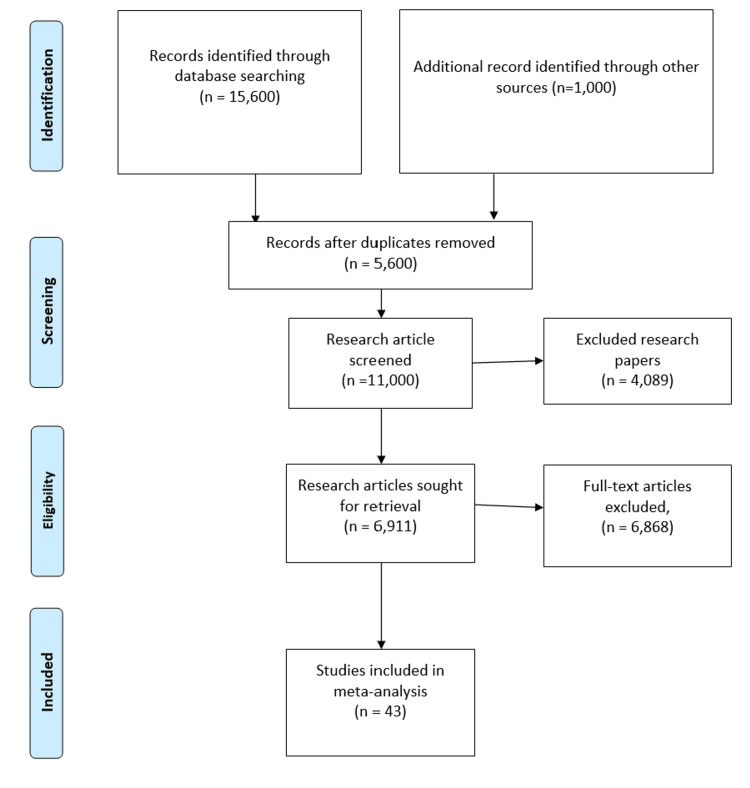
PRISMA flowchart outlining the screening and selection of included studies PRISMA: Preferred Reporting Items for Systematic Reviews and Meta-Analyses

The selection and screening of research articles related to the study aim, “Effectiveness of various biological therapies for treatment of UC,” was performed by following the PRISMA guidelines in this meta-analysis. A total of 39,000 research articles were extracted after applying the above-mentioned search strategy. Only 11,000 papers were initially screened, and 7,011 research articles were retrieved before final screening. Among those, only 3,001 articles were assessed for eligibility criteria, and the final number of research articles after applying exclusion criteria was 43, as mentioned in Figure 1. 

 Table 1 presents the characteristics of the included studies.

**Table 1:** Characteristics of the included studies Pre: measurement taken before an event (e.g., medication, procedure, exercise); Post: measurement taken after the event; T: test statistic; P: p-value; UC: ulcerative colitis

Author, year	Country	Study population (mean age in years)	Median follow-up	Study design	Drug used	Mucosal healing	Response rates	Clinical remission rates
Honap et al., 2020 [25]	United Kingdom	134 patients with UC (37 years)	26 weeks	Multicenter prospective cohort	Tofacitinib		Pre: 88/119, Post: 47/108	
Singh et al., 2024 [26]	India	104 patients with UC (37.5 years) T: 53 P: 51	90 days	Randomized controlled trial	Tofacitinib (10 mg thrice daily)		T: 44/53, P: 30/51	
Sandborn et al., 2017 [27]	United States	593 patients with UC (39.5 years), T: 197, P: 198	52 weeks	Phase 3, randomized-controlled study	Tofacitinib (10 mg thrice daily)		T; 122, P; 40	T: 80, P: 22
Panés et al., 2015 [28]	Spain	194 patients with UC, 3T: 31, P: 48	8 weeks	Phase 3, randomized-controlled study	Tofacitinib (10 mg thrice daily)		T: 16, P: 20	T: 12, P: 11
Ollech et al., 2024 [29]	Israel	30 adult patients (26.3 years)		Prospective real-world study	Tofacitinib	T: 10/30, P: 10/25		T: 12, P: 6
Hernández Martínez et al., 2022 [30]	Spain	74 patients (45.4 years), T: 40, P: 48	19 months	Retrospective and multicenter observational study	Tofacitinib		T: 24, P: 25	T: 28, P: 19
Sands et al., 2016 [31]	United States	1139 patients with UC (39.8 years), T: 905, P: 234	8 weeks	Randomized controlled trial	Tofacitinib 10 mg		T: 521, P: 72	T: 156, P: 14
Ma et al., 2023 [32]	Canada	334 patients with UC	52 weeks	Prospective cohort study	Tofacitinib 10 mg		T: 245, P: 109	T: 64/93, P: 106/300
Jameshorani et al., 2021 [33]	Iran	50 patients with UC (40.5 years)	52 weeks	Prospective cohort study	Tofacitinib 10 mg		T: 30, P: 19	
McNally et al., 2022 [34]	Ireland	53 UC patients (40.4 years)	6 months	Prospective cohort study	Tofacitinib 10 mg		T: 36, P: 22	
Sandborn et al., 2012 [35]	Canada	194 UC patients (42.5 years), T: 64, P: 48	8 weeks	Phase II, randomized controlled trial	Tofacitinib 10mg		T: 16, P: 5	T: 10, P: 1
Hong et al., 2020 [36]	United States	19 patients with UC (42.5 years)	12 months	Retrospective cohort study	Tofacitinib 10mg		T: 6, P: 4	T: 6, P: 10
Honap et al., 2022 [37]	United Kingdom	110 patients with UC (40 years)	28 weeks	Cohort study	6mg/kg of ustekinumab		T: 23/39, P: 32/55	T: 17/39, P: 21/55
Van Lierop et al., 2025 [38]	Canada	121 UC patients	141 weeks	Multicenter retrospective cohort study	6mg/kg of ustekinumab			T: 43/81, P: 22/40
Hong et al., 2021 [39]	USA	66 UC Patients (39.5 years)	12 months	Real-world study	90 mg subcutaneous (SC) injection of ustekinumab		T: 11/20, P: 23/47	T: 9/20, P: 20/47
Chiappetta et al., 2021 [40]	Italy	68 patients with UC (42 years)	52 weeks	Real-world study	6mg/kg of ustekinumab		Pre: 57, Post: 55	pre: 20, Post: 35
Tursi et al., 2024 [41]	Italy	256 patients with UC (52 years)	24 weeks	Retrospective, observational cohort study	6mg/kg of ustekinumab		T: 94/152, P: 42/152	T: 125/235, P: 44/235
Danese et al., 2019 [42]	Italy	642 patients with UC	16 weeks	Phase 3 randomized clinical trial	90 mg of ustekinumab		Pre: 169, post: 65	Pre: 40, post: 4
Parra et al., 2024 [43]	Brazil	56 UC patients (42.8 years)	52 weeks	Multicenter retrospective observational cohort study	90 mg of ustekinumab		Pre: 28/50, post: 34/50	Pre; 9/50, post: 25/47
Amiot et al., 2020 [44]	France	103 UC patients	16 weeks	GETAID multicenter real-world cohort study	90 mg of ustekinumab			Pre: 36, post: 40
Yarur et al., 2025 [45]	USA	245 UC patients	33 weeks	Multicenter real-world cohort study	90 mg of ustekinumab		T: 50, P: 29	T: 7/39, P: 15/63
Narula et al., 2018 [46]	Canada	321 UC patients	12 months	Retrospective cohort study	300 mg of vedolizumab		T: 70, P: 56	T: 64/321, P: 35/203
Sandborn et al., 2020 [47]	United States	216 UC patients (41.6 years), T: 106, P: 56	52 weeks	Phase 3, randomized controlled trial	300 mg of intravenous vedolizumab	T: 56, P: 21	T: 64, P: 28	T: 49, P: 8
Motoya et al., 2019 [48]	Japan	292 UC patients (44.6 years), T: 164 P: 82	10 weeks	Phase 3, randomized controlled trial	300 mg of intravenous vedolizumab	T: 60, P: 25	T: 65/164, P: 27/82	T: 30, P: 10
Loftus et al., 2016 [49]	USA	532 UC patients (41.7 years)	52 weeks	Prospective cohort study	300 mg of intravenous vedolizumab		T: 218, P: 148	T: 120/136, P: 70/73
Attauabi et al., 2021 [50]	Denmark	97 UC patients	52 weeks	Retrospective two-center cohort study	300 mg of intravenous vedolizumab			T: 35, P: 27
Feagan et al., 2017 [51]	Canada	UC patients (40.5 years) T: 464 P: 367	52 weeks	Prospective cohort study	300 mg of intravenous vedolizumab	T: 124, P: 22	T: 125, P: 27	T: 98, P: 17
Bosca-Watts et al., 2016 [52]	Spain	33 UC patients (40.4 years)	52 weeks	Prospective cohort study	50 mg of golimumab		T: 14, P: 6	T: 18 P:8
Eriksson et al., 2021 [53]	Sweden	50 UC patients (41 years)	52 weeks	Prospective cohort study	50 mg of golimumab		T: 14, P: 13	T: 8, P: 10
Bossa et al., 2020 [54]	Italy	196 UC patients	3 months	Prospective cohort study	50 mg of golimumab		T: 130, P: 53	
Tursi et al., 2017 [55]	Italy	93 UC patients (47.5 years)	6 months	Prospective cohort study	50-200 mg of golimumab		T: 72, P: 30	T: 16, P: 18
O’Connell et al., 2018 [56]	Ireland	72 UC patients (41.4 years)	6 months	Cohort study	50-200 mg of golimumab		T: 40, P: 32	T: 28, P: 18
Perrig et al., 2022 [57]	Switzerland	103 UC patients	1 year	Cohort study	50-200 mg of golimumab		T: 63, P: 51	T: 8, P: 52
Bressler et al., 2018 [58]	Canada	137 UC patients (44.4 years)	1 year	Cohort study	50-200 mg of golimumab		T: 105, P: 25	
Fumery et al., 2023 [59]	France	47 UC patients (39 years)	24 weeks	Prospective cohort study	50-200 mg of golimumab		T: 19, P: 15	T: 5, P: 10
Ogata et al., 2021 [60]	Japan	1,593 UC patients (41.8 years)	52 weeks	Prospective, multicenter, single-cohort,	Adalimumab	T: 1083, P: 971		T: 845, P: 165
García-Bosch et al., 2013 [61]	Spain	48 UC patients	54 weeks	Retrospective cohort study	Adalimumab	T: 40 P: 16		
Angelison et al., 2020 [62]	Sweden	118 UC patients (34.4 years)	1.27 years	Retrospective cohort study	Adalimumab		T: 91, P: 12	T: 38, P: 29
Suzuki et al., 2013 [63]	Japan	273 UC patients (42.5 years), T: 90, P: 96	52 weeks	Phase 2/3, randomized, double-blind study	Adalimumab	T: 40, P: 28	T: 45, P: 33	T: 10, P: 9
Armuzzi et al., 2013 [64]	Italy	88 UC patients (42.8 years)	54 weeks	Observational study	Adalimumab			T: 38, P: 15
Sandborn et al., 2012 [65]	United States	494 UC patients	52 weeks	Randomized, double-blind, placebo-controlled trial	40-160 mg adalimumab			T: 45/264, P: 20/230
Mohamed et al., 2017 [66]	Kuwait	48 adult patients with refractory UC (32.6 years)	12 weeks	Prospective cohort study	5 mg/kg of infliximab	T: 29, P: 15		
Tursi et al., 2017 [67]	Italy	29 UC patients (45 years)	24 weeks	Prospective cohort study	5 mg/kg of infliximab	T: 26, P: 29	T: 26, P: 14	T: 22, P: 29

Quality Assessment of the Included Studies

Among 33 observational cohort studies, 19 included studies were low risk, and 14 studies were moderate risk, as represented in Table 2. Most comparisons showed low to moderate evidence quality, and the study's limitations, inconsistencies, indirectness, and imprecision were the key reasons for the confidence decline.

**Table 2:** Quality assessment of the included studies by the Newcastle-Ottawa Scale *: Yes; 0: No

	Selection				Comparability		Outcome			
Study	Representative of the exposed cohort	Selection of external control	Ascertainment of exposure	Outcome of interest not present	Main factor	Additional factor	Assessment of outcome	Sufficient follow-up time	Adequacy of follow-up time	Total
Honap et al., 2020 [25]	*	0	*	0	*	0	*	*	*	6/9
Ollech et al., 2024 [29]	*	*	*	0	*	0	*	0	0	5/9
Hernández Martínez et al., 2022 [30]	*	*	0	*	*	*	*	0	*	7/9
Ma et al., 2023 [32]	*	*	0	*	*	0	*	0	0	5/9
Jameshorani et al., 2021 [33]	*	0	*	0	*	*	*	*	*	7/9
McNally et al., 2022 [34]	*	*	*	0	*	0	*	0	0	5/9
Hong et al., 2020 [36]	*	*	0	*	*	*	*	8	*	8/9
Honap et al., 2022 [37]	*	*	*	0	*	0	*	0	0	5/9
Van Lierop et al., 2025 [38]	*	*	0	*	*	*	*	0	*	7/9
Hong et al., 2021 [39]	*	*	0	*	*	0	*	0	0	5/9
Chiappetta et al., 2021 [40]	*	0	*	0	*	*	*	*	*	7/9
Tursi et al., 2024 [41]	*	0	*	0	*	*	*	*	*	7/9
Parra et al., 2024 [43]	*	0	*	0	*	0	*	*	*	6/9
Amiot et al., 2020 [44]	*	0	*	0	*	0	*	*	*	6/9
Yarur et al., 2025 [45]	*	*	*	0	*	0	*	0	0	5/9
Narula et al., 2018 [46]	*	*	0	*	*	*	*	0	*	7/9
Loftus et al., 2016 [49]	*	*	0	*	*	0	*	0	0	5/9
Attauabi et al., 2021 [50]	*	0	*	0	*	*	*	*	*	7/9
Feagan et al., 2017 [51]	*	*	*	0	*	0	*	0	0	5/9
Bosca-Watts et al., 2016 [52]	*	*	0	*	*	*	*	8	*	8/9
Eriksson et al., 2021 [53]	*	*	*	0	*	0	*	0	0	5/9
Bossa et al., 2020 [54]	*	*	0	*	*	*	*	0	*	7/9
Tursi et al., 2017 [55]	*	*	0	*	*	0	*	0	0	5/9
O’Connell et al., 2018 [56]	*	0	*	0	*	*	*	*	*	7/9
Perrig et al., 2022 [57]	*	0	*	0	*	*	*	*	*	7/9
Bressler et al., 2018 [58]	*	0	*	0	*	0	*	*	*	6/9
Fumery et al., 2023 [59]	*	0	*	0	*	0	*	*	*	6/9
Ogata et al., 2021 [60]	*	*	*	0	*	0	*	0	0	5/9
García-Bosch et al., 2013 [61]	*	*	0	*	*	*	*	0	*	7/9
Angelison et al., 2020 [62]	*	*	0	*	*	0	*	0	0	5/9
Mohamed et al., 2017 [66]	*	*	0	*	*	*	*	0	*	7/9
Tursi et al., 2017 [67]	*	*	0	*	*	0	*	0	0	5/9
Armuzzi et al., 2013 [64]	*	*	0	*	*	*	*	0	*	7/9

Risk of Bias Assessment

**Figure 2:**
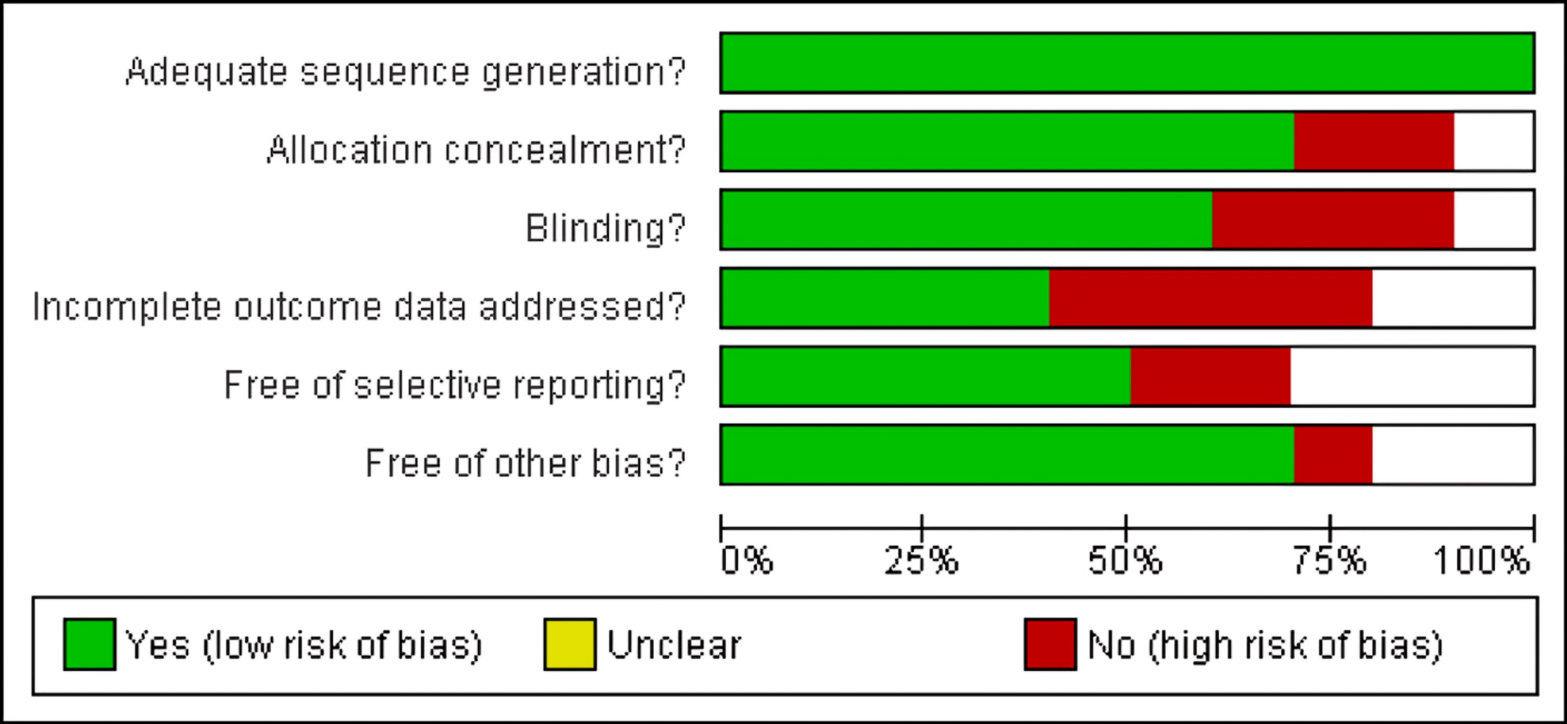
Representation of the risk bias of the included studies

**Figure 3:**
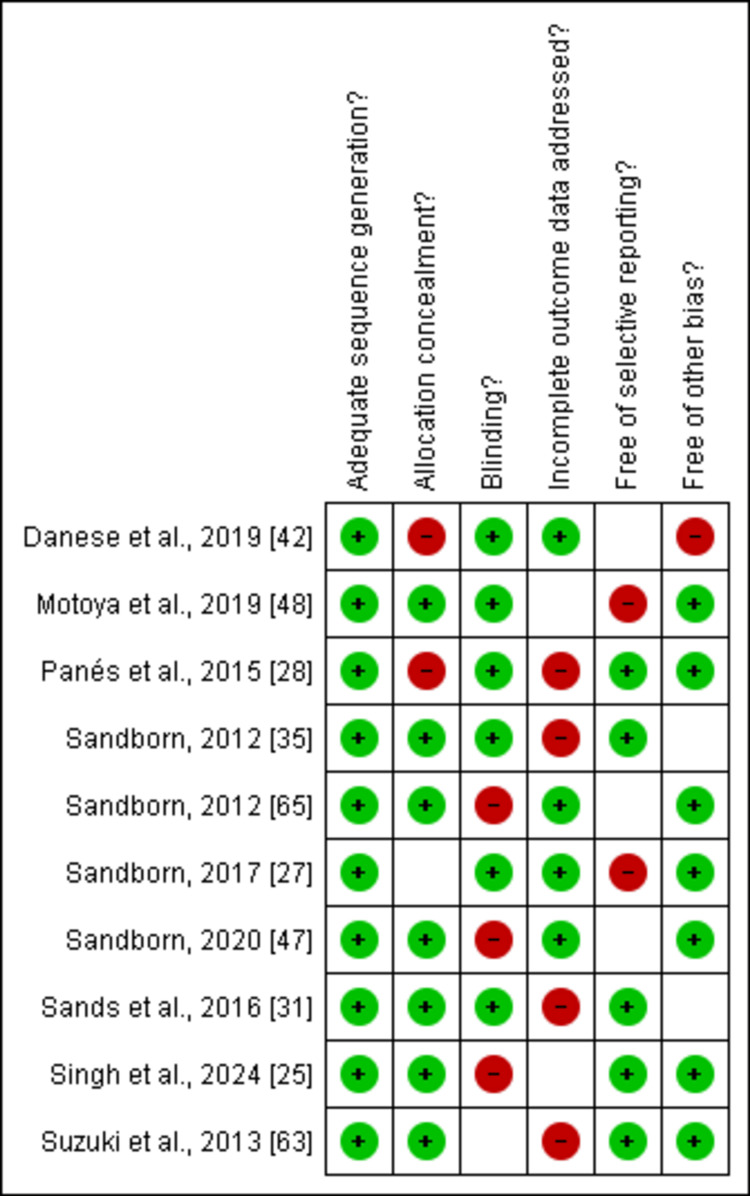
Representations of the risk bias summary of the included studies Studies included: [25, 27, 28, 31, 35, 42, 47, 48, 63, 65]

The Cochrane tool was used for risk bias assessment of the 10 included RCTs. All 10 included studies were low risk, as mentioned in Figure 2 and Figure 3.

Primary Outcomes

**Figure 4:**
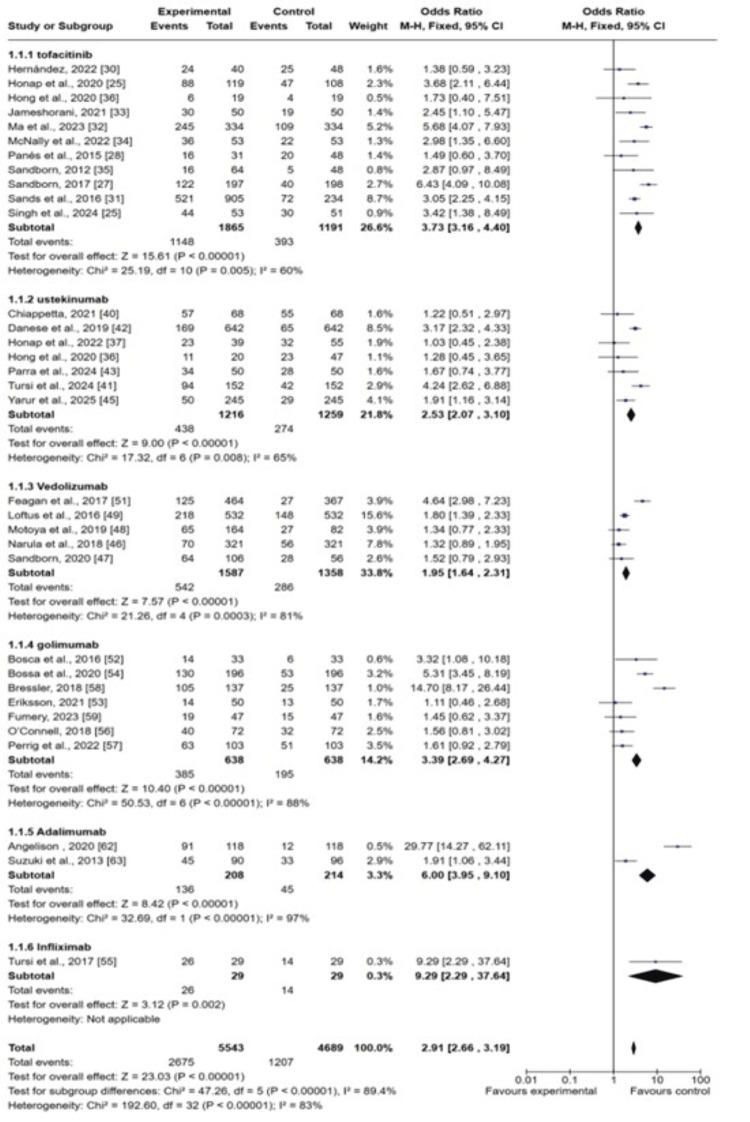
Forest plot of the odds ratio of response rates among UC patients receiving biological drugs as compared to placebo CI: confidence interval; UC: ulcerative colitis 1.1.1: [25, 27-28, 30-36, 40]; 1.1.2: [37, 36, 41-43, 45]; 1.1.3: [46, 47, 48, 49, 51]; 1.1.4: [52-54, 57, 58, 66, 68, 69]; 1.1.5: [62, 63]; 1.1.6: [55]

Response rates: Among 43 included studies, 33 research studies have reported the response rates as outcomes in UC patients receiving treatment with biological drugs as compared to placebo. The pooled analysis showed that clinical response rates improved among UC patients after receiving biological drugs as compared to placebo (odds ratio (OR): 2.19 (CI 95%: 2.66-3.19), p<0.00001, I2= 83%), as shown in Figure 4.

**Figure 5:**
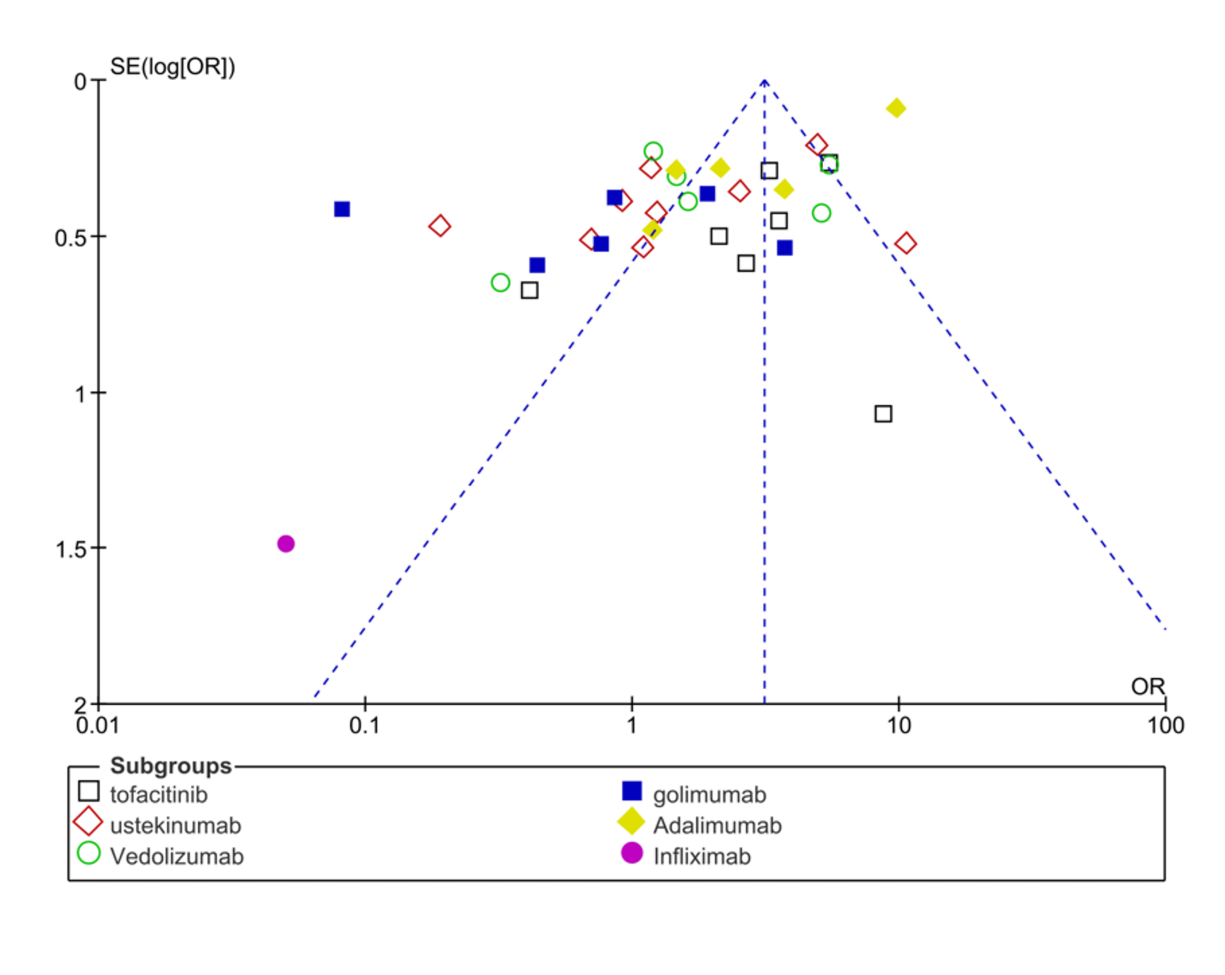
Funnel plot of the odds ratio (OR) of response rates among UC patients receiving biological drugs as compared to placebo UC: ulcerative colitis Studies included: [25, 27, 28, 31, 35, 42, 47, 48, 63, 65]

The subgroup analysis of different biological drugs showed that tofacitinib is more effective in improving response rates (OR: 3.73 (95% CI; 3.16-4.40), p<0.0001) and vedolizumab is least effective in terms of improving response rates (OR: 1.95 (1.64-2.33), p<0.00001). The symmetrical distribution of studies on the funnel plot showed the low publication bias as reported by meta-regression results of response rates among the included studies (Figure 5). 

**Figure 6:**
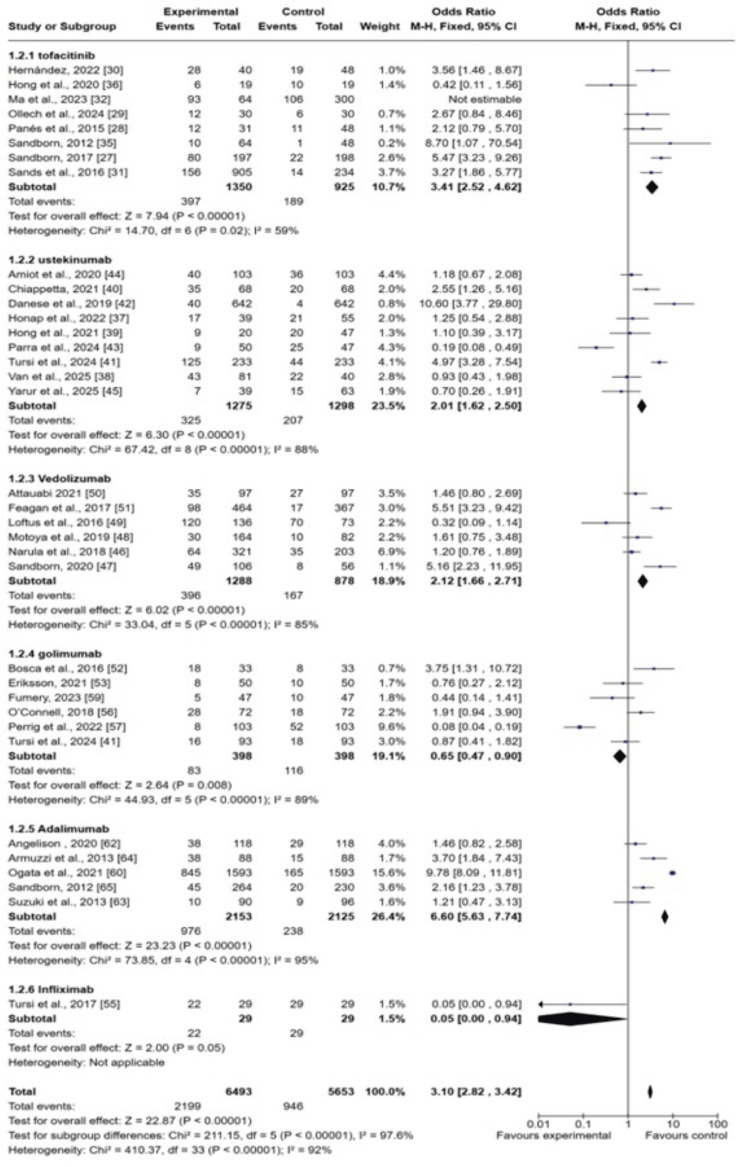
Forest plot of the odds ratio of remission rates among UC patients receiving biological drugs as compared to placebo CI: confidence interval; UC: ulcerative colitis 1.2.1: [27-32, 35-36]; 1.2.2: [37-39, 40-45]; 1.2.3: [46-51]; 1.2.4 [41, 52-53, 56-57, 59]; 1.2.5: [60, 62-65]; 1.2.6: [55]

Remission rates: Among 43 included studies, 37 research studies have reported the clinical remission rates as outcomes in UC patients receiving treatment with biological drugs as compared to placebo. The pooled analysis showed that clinical response rates improved among UC patients after receiving biological drugs as compared to placebo (OR: 3.10 (CI 95%: 2.82-3.42), p<0.00001, I2= 92%), as shown in Figure 6.

**Figure 7:**
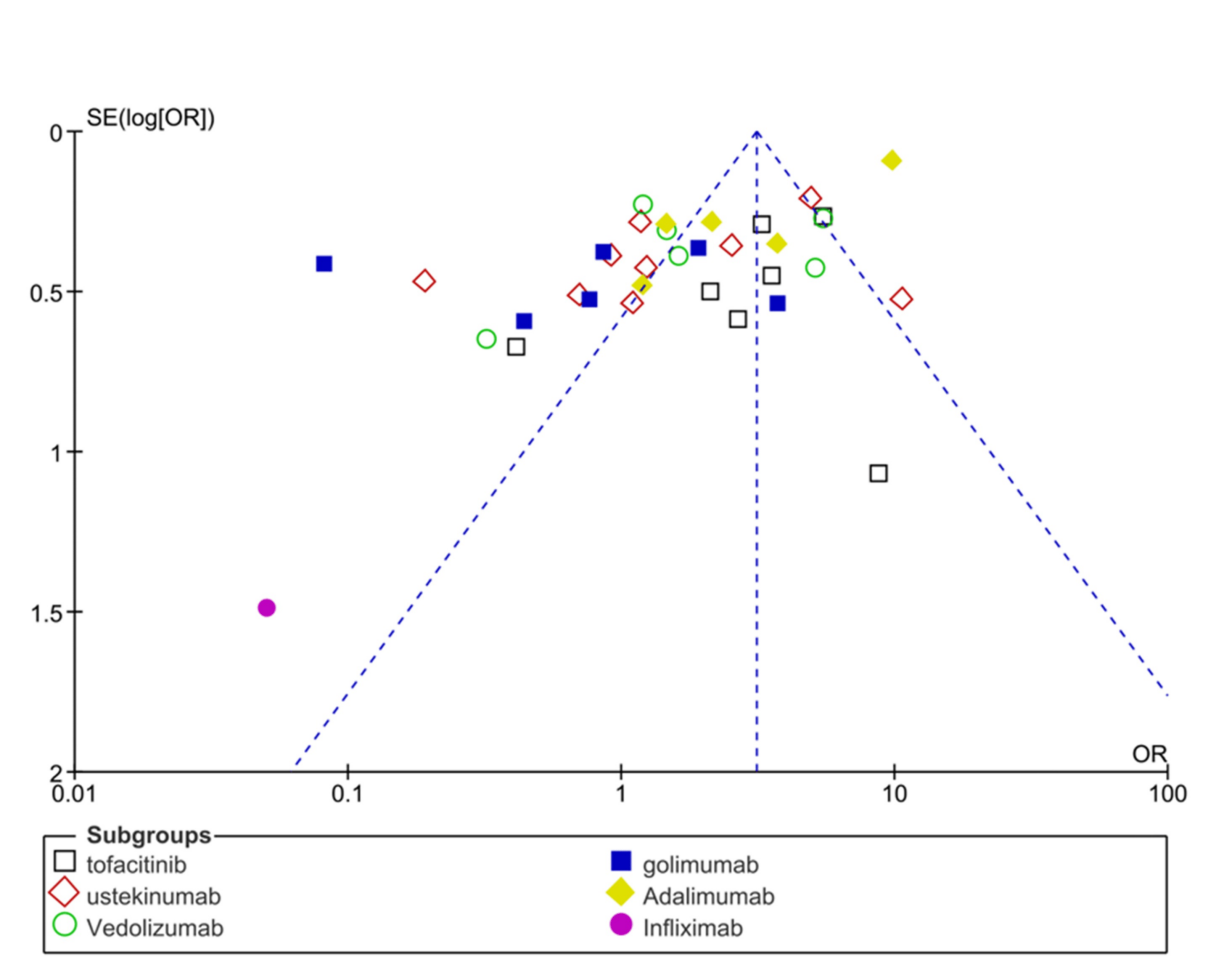
Funnel plot of the odds ratio (OR) of remission rates among UC patients receiving biological drugs as compared to placebo UC: ulcerative colitis Studies includes: [25, 27, 28, 31, 35, 42, 47, 48, 63, 65]

The subgroup analysis of different biological drugs showed that adalimumab is more effective in improving remission rates (OR: 6.60 (95% CI; 5.63-7.74), p<0.0001) and golimumab is least effective in terms of improving remission rates (OR: 0.65 (0.47-0.90), p<0.00001). The symmetrical distribution of studies on the funnel plot showed the low publication bias as reported by meta-regression results of response rates among included studies, as shown in Figure 7.

**Figure 8:**
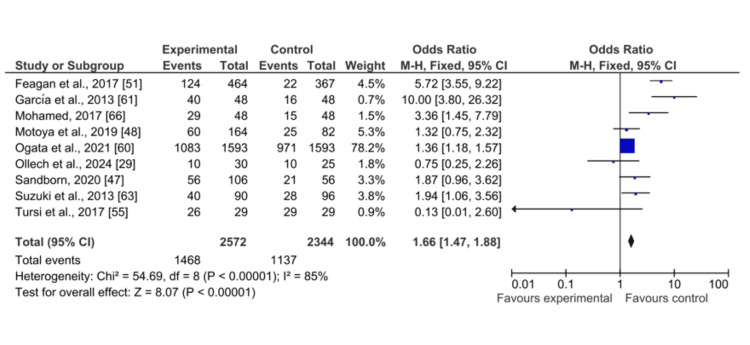
Forest plot of the odds ratio of mucosal healing among UC patients receiving biological drugs as compared to placebo CI: confidence interval; UC: ulcerative colitis Studies included: [29, 47, 48, 51, 55, 60, 61, 63, 66]

Among 43 included studies, only nine research studies have reported the clinical mucosal healing as an outcome in UC patients receiving treatment with biological drugs as compared to placebo. The pooled analysis showed that mucosal healing significantly improved among UC patients after receiving biological drugs as compared to placebo (OR: 1.66 (CI 95%: 1.47-1.88), p<0.00001, I2= 85%) as shown in Figure 8.

**Figure 9:**
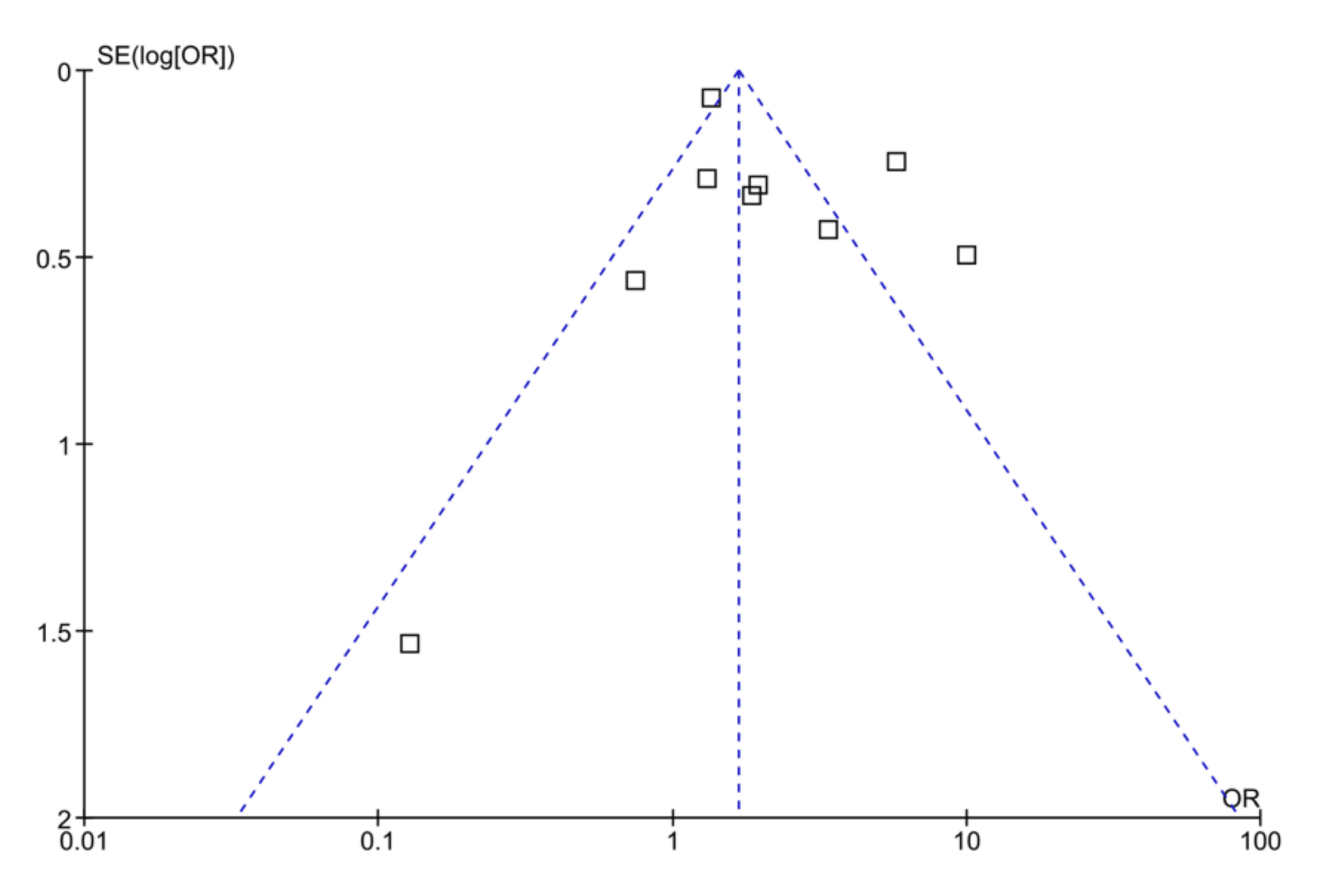
Funnel plot of the odds ratio (OR) of mucosal healing among UC patients receiving biological drugs as compared to placebo UC: ulcerative colitis Studies included: [25, 27,  28, 31, 35, 42, 47, 48, 63, 65]

The symmetrical distribution of studies on the funnel plot showed the low publication bias as reported by the meta-regression results of response rates among included studies, as shown in Figure 9.

Discussion

This systematic review and meta-analysis evaluated the efficacy of six biological drugs in treating UC across 43 included studies. The results demonstrated that biological drugs collectively outperform placebo across all key clinical outcomes. Patients receiving biological therapies showed significantly higher response rates (OR: 2.19, 95% CI: 2.66-3.19*), remission rates (OR: 3.10, 95% CI: 2.82-3.42), and mucosal healing rates (OR: 1.66, 95% CI: 1.47-1.88), with all outcomes achieving high statistical significance (p < 0.00001). Despite substantial heterogeneity across studies (I² = 83-92%), consistent benefits were observed. All RCTs included were assessed as low-risk using the Cochrane bias assessment tool, strengthening the reliability of these findings. While 33 cohort studies were assessed by NOS, 19 studies were predicted to be low risk, and 14 studies were predicted to be moderate risk. The symmetrical distribution of studies on the funnel plot showed low to moderate publication bias among included studies. 

The findings of this study were consistent with previous studies that reported the effectiveness and safety of biologics and small-molecule drugs for the treatment of UC patients [68-70]. These studies proved that biological agents are effective treatments for UC patients with fewer adverse events as compared to other therapeutic strategies.

This meta-analysis has several strengths. It assessed 43 studies from six varied biological therapies in a complete manner, thus giving a general overview of their efficacy for UC treatment. The use of systematic review and meta-analysis methodology increases the validity of the findings by aggregating data from RCTs and observational cohort studies. The use of strict quality assessment measures, Cochrane for RCTs and NOS for cohort studies, prevented methodological bias, as the majority of studies were classed as low risk. The statistically significant pooled odds ratios for clinical response, remission, and mucosal healing, each with p-values < 0.00001, strongly indicate that biological therapies work in moderate-to-severe UC. In addition, the symmetrical shape of the funnel plot indicates low publication bias, validating the findings.

Nonetheless, a number of limitations need to be recognized. The high heterogeneity between studies (I² of 83% to 92%) can affect the consistency of the pooled estimates and reflects variation in study populations, interventions, and outcome measures. While the majority of studies were at low to moderate risk, the inclusion of observational cohort studies may introduce potential confounding and selection bias. Study design differences, differing follow-up lengths, and differing drug dosing regimens might also have contributed to differences in the findings. Publication bias seemed low but cannot be entirely eliminated, particularly since biologic drug trials are of high-profile status and possibly receive industry sponsorship.

Clinically, the findings highlight the key position biologics play in the therapeutic management of moderate-to-severe UC. Clinicians can consider using biologics not only for clinical response induction but also for remission and mucosal healing, which are key to the patient's improved quality of life and long-term outcomes. The findings are consistent with existing guidelines and are likely to contribute to therapeutic decision-making, especially among patients with refractoriness to conventional therapies.

## Conclusions

Overall, this study offers robust evidence that biological treatments substantially enhance treatment outcomes in patients with moderate-to-severe UC over placebo. Although there is some heterogeneity and limitations in combining studies of different types, the overall quality of evidence is low to moderate. These findings support the clinical utility of biologics and underscore the importance of ongoing comparative studies and long-term outcome data to further refine treatment strategies.
